# Patient Acceptance, Ease of Use, and Preference for Norditropin NordiFlex with NordiFlex PenMate: Results from an Open-Label, User Survey of Everyday Use

**DOI:** 10.5402/2011/803948

**Published:** 2011-09-20

**Authors:** Anita Hokken-Koelega, Alexandra Keller, Viatcheslav Rakov, Stefan Kipper, Jovanna Dahlgren

**Affiliations:** ^1^Department of Paediatrics, Division of Endocrinology, Sophia Children's Hospital/Erasmus University Medical Centre, 3015 GJ Rotterdam, The Netherlands; ^2^Kinderzentrum am Johannis Platz, 04103 Leipzig, Germany; ^3^Biopharm Medical Affairs, Novo Nordisk Health Care AG, Andreasstrasse 15, Oerlikon 8050, Zurich, Switzerland; ^4^Clinical Research Department, Novo Nordisk Pharma GmbH, 55127 Mainz, Germany; ^5^Goteborg Pediatric Growth Research Center, Institute for Clinical Sciences, Sahlgrenska Academy at Goteborg University, SE41345 Goteborg, Sweden

## Abstract

In this 12-week open-label, uncontrolled study, patients (*n* = 85; mean [SD] age 11.2 [3.95] years) were trained to use an injection device with an automatic needle insertion accessory (NordiFlex/NordiFlex PenMate: Novo Nordisk A/S, Bagsvaerd, Denmark) for growth hormone (GH) injection. The opinions of patients and the physicians/nurses who trained patients on device were recorded by questionnaire. Most (88.4%) patients reported that the device was “very easy/easy" to use. The majority (82.4%) of patients were “very satisfied/satisfied" with the device and 64% wished to continue its use. Device training instructions were reported as “very easy/easy" by 96.1% of physicians/nurses, and 65.8% of participants could use the device after ≤10-minute training. In this study, NordiFlex PenMate was well accepted by patients and medical staff. Patients had a high opinion of the device and over half wished to continue its use. High patient acceptance may facilitate treatment adherence optimizing treatment outcomes.

## 1. Introduction

Since its development in 1985, recombinant human growth hormone (GH) has been widely available for the treatment in childhood of many indications, including growth hormone deficiency (GHD), Turner syndrome, chronic renal insufficiency, and short children born small for gestational age [1]. The majority of patients, especially those treated from an early age, respond well to treatment, achieving an adult height that is within the target range [2, 3]. Treatment success is dependent, however, on full adherence with the prescribed GH treatment regimen. If not well adhered to, improvements in linear growth may be impaired [4, 5]. GH products are administered daily by subcutaneous injection, and this may be associated with problems of adherence [5]. Given that GH treatment is administered daily by subcutaneous injection, it is important that the injection devices used are well accepted by patients, simple to use, easy to prepare, and simple to learn. Moreover, since a high proportion of those treated are young children, the device should be child-friendly and well accepted by the parents or caregivers who may be responsible for treatment administration. Although the first GH injections were administered using a traditional needle and syringe, advances in technology have seen the introduction of devices that vary the method of subcutaneous injection and the injection product [6]. 

In subjective evaluations of patient preferences, ease of use and lack of pain during injections are found to rank among the top five device attributes of delivery devices for administering GH [7, 8]. The appearance or visibility of the needle is psychologically difficult for many younger patients, and for this reason they often rely on adults to perform their injections [9]. 

Pen injection devices, the current “gold standard” for injection, are associated with improved convenience and reduced pain on injection and allow a larger proportion of children to inject themselves [6]. Improving the ease of injection and reducing the pain associated with injection has the potential to increase compliance with therapy [10].

NordiFlex (Novo Nordisk A/S, Bagsvaerd, Denmark) is a prefilled, multidose, disposable pen injection device containing liquid GH (Norditropin SimpleXx, Novo Nordisk A/S, Bagsvaerd, Denmark). NordiFlex PenMate is an automatic needle insertion device, specifically for use with NordiFlex, designed to ease injection pain and reduce needle fear. The NordiFlex PenMate design may positively contribute to compliance [10]. In children with diabetes, use of PenMate with the insulin injection pen NovoPen (Novo Nordisk A/S, Bagsvaerd, Denmark) was shown to be associated with lower perception of pain [11]. 

In this study, we assessed patient acceptance and compliance with NordiFlex PenMate in usual clinical practice in children currently on, or starting, GH therapy. Assessment of patient acceptance was done by questionnaire delivered at the end of a 12-week period.

## 2. Methods

### 2.1. Patients

Patients who attended outpatient pediatric clinics at 16 sites (11 in Germany, four in Sweden, and one in the Netherlands) between January 2007 and March 2009 who were prescribed NordiFlex in combination with NordiFlex PenMate as part of usual clinical practice were eligible for inclusion in the study. Inclusion criteria were that the patients should be eligible for treatment with NordiFlex in accordance with locally approved labeling. Patients could be naïve to GH therapy or currently on GH therapy (excluding NordiFlex) with another GH product but required switching to a new device. Children with contraindications to treatment with Norditropin or who had previously been treated with NordiFlex were excluded from the study. Study-specific written informed consent was obtained in accordance with local guidance. The selection of patients for inclusion in the study was at the discretion of the individual treating physicians.

A total of 85 patients (55% boys; Germany, *n* = 21; The Netherlands, *n* = 49; Sweden, *n* = 15) was included in the study. Patients (and, if appropriate, their parents or guardians) were given training in the use of NordiFlex with NordiFlex PenMate. Either the patients or their parents used the device to administer GH subcutaneously in the evening. The daily starting dose and frequency, as well as any change in dose or frequency, were calculated by the treating physician. Patients were issued with diaries at the first visit.

The primary endpoint of the study was the proportion of patients who found NordiFlex with NordiFlex PenMate “very easy” or “easy” to use after 12 weeks of treatment. Secondary endpoints included ease of teaching the device, assessed by nurse questionnaire at the start of the study, patient and parent overall acceptance of the device system and preference to use NordiFlex with NordiFlex PenMate compared to previous device use, where appropriate. 

**Figure 1:**
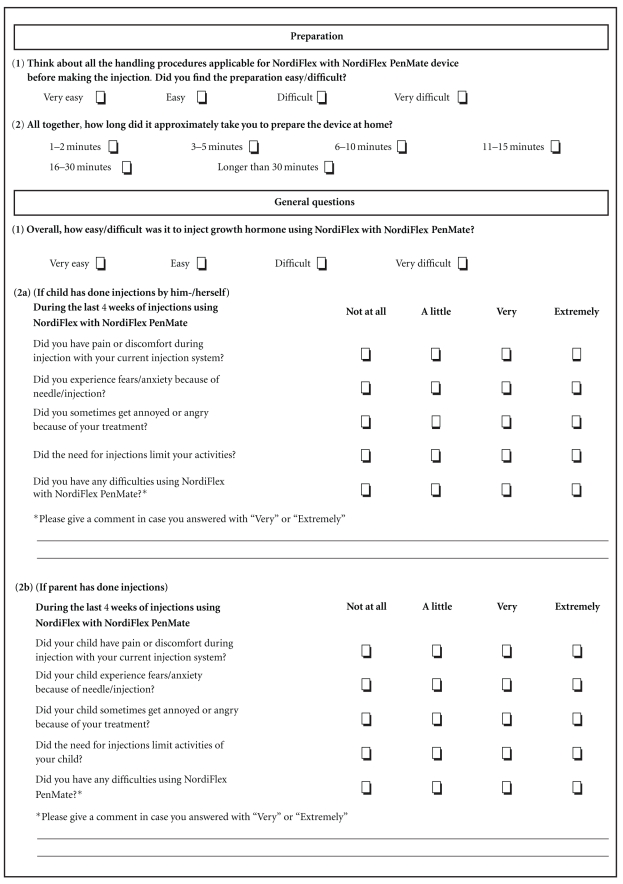

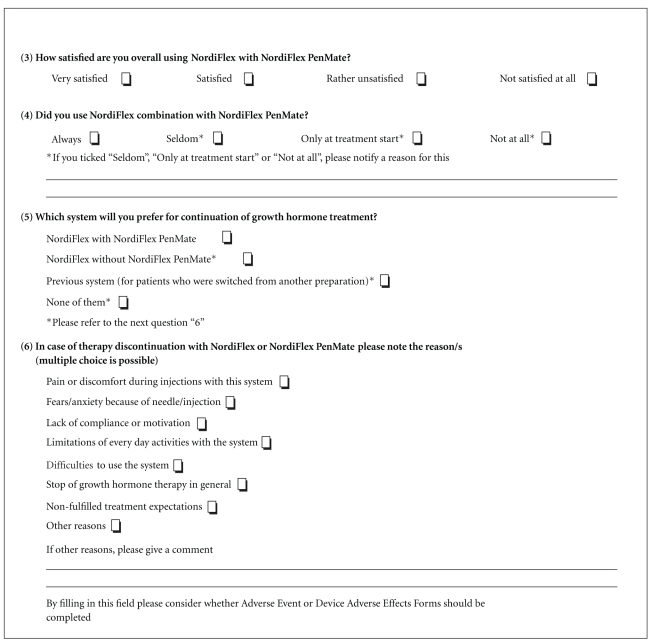
Questionnaire used to assess patients' acceptance of the Norditropin NordiFlex pen and NordiFlex PenMate in pediatric patients treated with growth hormone.

Data were collected at baseline and at the end of the observation period of 12 weeks (mean 17(±7) weeks) after treatment start. Baseline assessments included anthropometry (height, weight, body mass index, and pubertal status). At baseline, the physician or nurse completed a brief questionnaire that assessed training time and ease of training in the device. At the end of the observation period, at a routine clinic visit, the physician or responsible study personnel determined the patients' and parents' impressions of the device using a six-item questionnaire (Figure 1). The questionnaire was subdivided into two sections on preparation (ease and time needed to prepare the pen for injection), plus general questions. In the general section, question 1 assessed the difficulty of the injection, question 2 assessed the patients' comfort with the injection (depending on whether they were self-injecting or their parents/guardians performed the injection), and question 3 evaluated patient satisfaction. In question 4, patients were asked if they preferred to use NordiFlex with or without NordiFlex PenMate and question 5 assessed patients' comfort with the idea of future use of NordiFlex PenMate. Question 6 assessed technical difficulties with the device during use. Compliance with treatment was assessed by patient diary. Safety was assessed through the recording of adverse events.

### 2.2. Data Analysis

Data are shown for the whole patient population. Standard descriptive statistics were used. The primary endpoint was the proportion of subjects who found NordiFlex with Norditropin PenMate “very easy” or “easy” to use. The proportion was summarized using frequency count. Ease of teaching and learning, other acceptability variables, and compliance to treatment were also assessed using frequency count. Mean (SD) values were calculated for the baseline characteristics like patients' age, height, weight, and body mass index. As is typical in observational trials using data from patient questionnaires, data were not available from all participants for all questions. Furthermore, in some cases, both the patient and his/her caregiver gave responses to questions. Since the omission of any data was assumed to be random, there was no input for missing data and all responses have been reported. Thus, for each question, the base population is dependent on the number of questionnaires returned with an answer for that particular question.

## 3. Results

Patient demographic characteristics at baseline are shown in Table 1. Mean (SD) age in children who self-injected was 13.7 (2.2) years, which was older than the mean age for patients whose parents performed the injection (9.0 (3.8) years) and for those where injections were performed by both patients and their parents (10.3 (3.2) years). Of patients (*n* = 66) who switched from other GH injection devices, 10 (15%) changed device due to fears/anxieties associated with needles or the injection process, one patient due to lack of compliance, and another due to difficulties with their existing device. No reason was given for other patients switching to NordiFlex.

A total of 12 patients discontinued treatment with NordiFlex PenMate during the observation period. Reasons given for discontinuation were as follows: four (5%) stopped due to pain or discomfort, three (4%) because of difficulties with device use, two (2.5%) because they had finished treatment with GH, two (2.5%) due to “other” reasons, and one (1%) patient each because of lack of compliance, fears/anxieties, limitations, and nonfulfilled expectations (note that one patient reported three reasons and another gave two reasons). Overall, 79-patient questionnaires were completed and returned for analysis and 22-patient diaries (10 filled out by children, 12 completed by parents) were completed and returned.

### 3.1. Efficacy Evaluation

**Figure 2:**
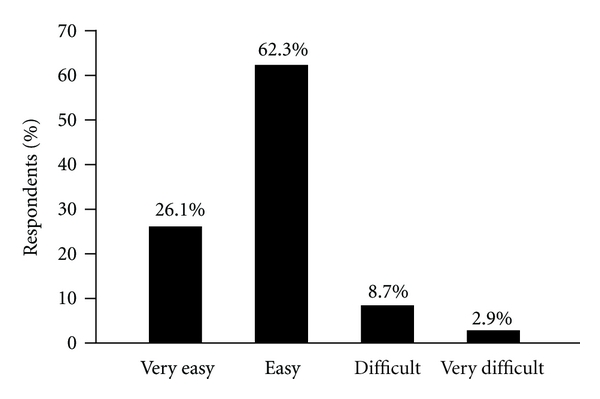
Patients' assessment of treatment with NordiFlex with NordiFlex PenMate at the end of the observation period (mean 17 ± 7 weeks) (*n* = 79).

During the observation period, 88.4% (61/69) of children reported that treatment with NordiFlex PenMate was “very easy” or “easy” (Figure 2). Few patients (11.6%, 8/69) reported that the injection process was “difficult” or “very difficult”. “No difficulties” with the injection process were reported by 90% (27/30) of children who self-injected, by 88.5% (23/26) of caregivers who performed the injections, and by 83.4% (10/12) of patients who were injected by themselves or their caregivers. Patients naïve to GH treatment (*n* = 16) rated the easiness of injections even more positively than those on established treatment regimens, with 93.8% (15/16) rating the injection process “easy” or “very easy”.

Patients' overall impression of the device was good, with the majority of patients (82.4%, 56/68) reporting that they were “satisfied” or “very satisfied” with the device; only 5.9% (4/68) were “not satisfied”. All treatment-naïve patients reported that they were “satisfied” or “very satisfied”. For caregivers who injected children, 86% (24/28) reported satisfaction with the device, while 82.1% (23/28) of children who self-injected stated that they were “satisfied” with the device. In families where both patients and caregivers performed injections, 75% (9/12) were satisfied with treatment. All patients who reported that they were “rather unsatisfied” or “not satisfied” with the device (*n* = 12) had been switched from other devices.

Over half of respondents (64%, 46/72) reported that they would prefer to use NordiFlex PenMate for future GH delivery. Of the remaining patients, 18% (13/72) reported that they would like to use NordiFlex without PenMate in the future, while a similar proportion (18%) preferred their original device over NordiFlex PenMate. Preference for future use of NordiFlex PenMate was especially high in patients who were treatment-naïve (80%) and in caregivers who performed the injections (74.1%). 

Almost all patients (97.4%, 75/77) found preparation of NordiFlex PenMate for injection “very easy” or “easy”, with only two patients stating that they found the process difficult. In almost two-thirds of patients (62.2%, 46/74) the injection preparation time was less than 2 minutes, and it was less than 5 minutes for all but two of the remaining patients. The latter two patients were able to prepare the device for injection in less than 10 minutes.

**Figure 3:**
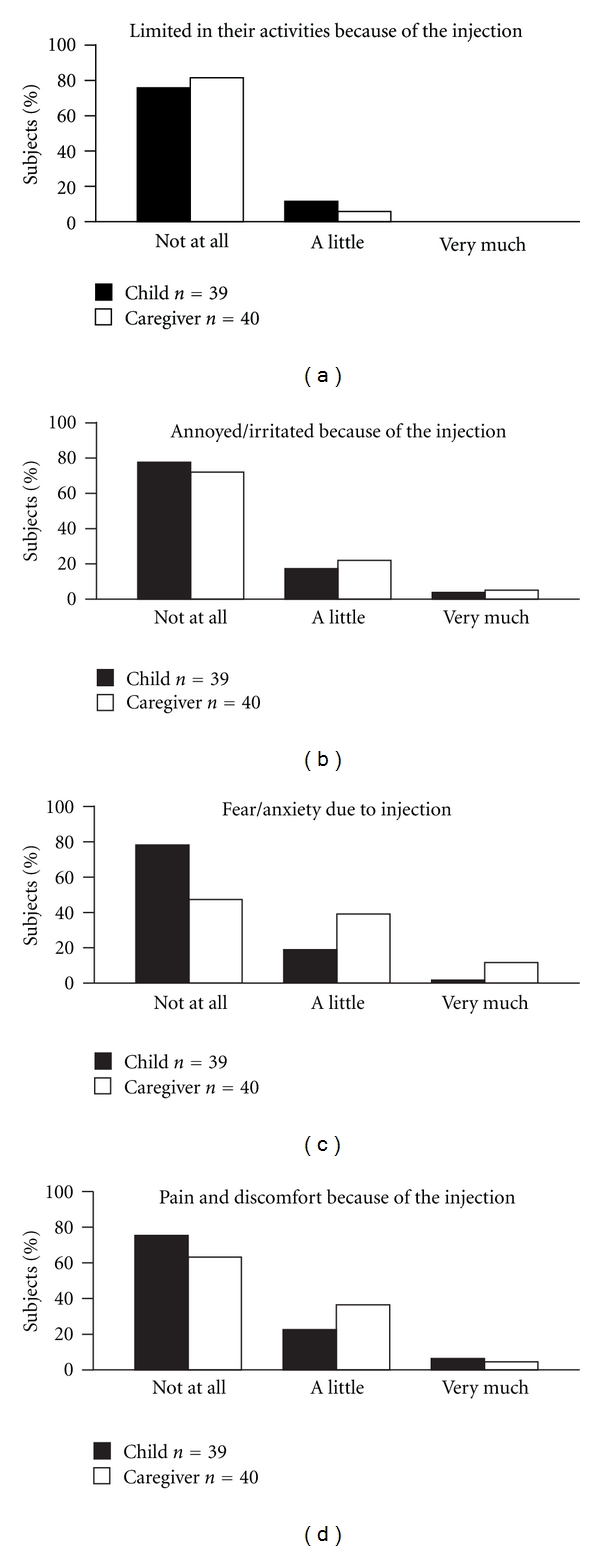
Patients' rating of the injection process using NordiFlex with NordiFlex PenMate, rated according to whether the injection was performed by the child (self-injection) (*n* = 39) or by his/her caregiver (*n* = 40). In both groups, a small number of families gave responses from both the child and caregiver; therefore, a minor overlap in responses was observed.

Most patients reported very little pain on injection and little fear or anxiety with the injection process (Figure 3). The use of the device was not considered to have a significant adverse effect on patients' activities or to cause any annoyance or irritation due to its use. Although the ratings were generally not that different when injections were performed by the child or by the caregiver, patients identified slightly more anxiety and pain or discomfort when the injections were not given by themselves.

Data from the returned patient diaries (12 completed by caregivers and 10 completed by self-injecting children) indicated that overall compliance was good in the observed cohort. Among children who self-injected, on average, 3.4 injections/child were missed during the observation period, and among caregivers, there was an average of 2.1 injections/patient missed during this period. None of the injections were missed due to fear/anxiety about the injection process; most were missed as they were either forgotten, the device was not available at the time of injection, or for other reasons.

### 3.2. Nurses, and Physicians' Opinions of Device Training

Instruction in device use was given to 30 caregivers, 15 patients and 38 patients in association with caregivers. The majority of physicians (96.1%) who trained participants in the use of the device reported that the device instructions were “very easy” or “easy”. Initial training in the device took 6–10 minutes for 40.2% (33/82) of patients or their caregivers and 3–5 minutes for 25.6% (21/82) of patients or caregivers. Overall, only 31.7% of patients required 10 minutes or more of initial training. For patients switching from other devices, 87.5% required 10 minutes or less of instruction.

### 3.3. Safety Results

One child had a rash (nonserious adverse drug reaction) and three reported technical complaints (needle placement (two reports) and uncertainty about the correct dosing (one report)) during the observation period.

## 4. Discussion

In the current study, patients and, where appropriate, their caregivers, as well as the nurses who provided training, had a high opinion of NordiFlex PenMate. After the observation period, almost 90% of patients reported that injection of GH using NordiFlex PenMate was “very easy” or “easy”; few patients reported any difficulties using the device. In line with these observations, high patient satisfaction with the device was reported by both children who self-injected and children who had their injections given by caregivers. It is noteworthy that treatment satisfaction was especially high among treatment-naïve patients. Indeed, after the observation period, most patients (80%) stated that they would like to continue using the device in the future. 

Patients and their caregivers quickly learned to use the device and found the injection process easy to learn. Initial training was completed in less than 10 minutes for two-thirds of patients and caregivers. Training time was further reduced in patients switching from other devices. Almost all of the physicians who provided training found the instructions for use “very easy” or “easy” to deliver. In support of these findings was the observation that half of the patients were able to self-inject without assistance. Ease of use is recognized as a key factor in device acceptance [8] and is especially important when the device is to be used by a child or adolescent. Key factors in ease of use are the number of steps in device preparation (the fewer the better) and ease of dose setting. In the present study, more than half of the patients were able to prepare the device for injection in less than 2 minutes and almost all patients found the device “easy” or “very easy” to prepare for use. Ease of use might also increase the number of patients who self-inject, which has been found to have a positive effect on treatment adherence [12]. In a multicenter questionnaire survey involving patients and/or their relatives who had been on GH treatment for more than 6 months, patients who self-injected GH were reported to have a significantly higher rate of adherence than those who had the injection administered by a parent, guardian, or healthcare professional (*P* < 0.01) [12]. 

Pain and perceived pain associated with injection may directly affect the patient's adherence and acceptance of treatment; hence, it is important to minimize pain associated with the injection process. Both injection technique and needle quality may influence injection pain [13]. Reduced needle diameter has been shown to have a marked impact on the reduction of injection pain [14], and previous studies have shown that patients prefer autoinjection over manual insertion of a needle [15, 16]. A key aim of the NordiFlex PenMate design is to make the injection process as easy and painless as possible. In 57 patients with diabetes, the use of NordiFlex PenMate was associated with a significant (*P* < 0.05) reduction in pain on injection [11]. In the present study, a lack of pain with injection was reported by 71.7% of patients who self-injected and by 60% of those whose injections were given by their parents. Overall, only four patients stated that they experienced significant pain on injection. 

While the experience of pain undoubtedly has a significant impact on patients' experience with a GH injection device, the ability to carry out activities of daily life without interference from therapy could also influence their impression of the device. When asked to consider factors such as discomfort, anxiety, annoyance or anger, and feelings of being limited in activities, most patients responded positively, indicating that they considered the device to have little impact on their activities of daily living.

Growth velocity is directly influenced by both the dose of GH and the frequency of injection [3]. Children with poor adherence have significantly reduced growth rates compared to those who receive a higher proportion of their prescribed dose [4, 17]. A questionnaire study of 75 GH-deficient children revealed that missing two or more injections per week was associated with a 42% reduction in growth velocity compared with children who received all their weekly injections (4.6 cm/year versus 7.8 cm/year; *P* < 0.05). In that study, 39% missed more than one injection per week and 23% missed more than two injections per week [17]. These proportions are consistent with other studies using objective assessments. Hunter et al. found that 33% of patients (<18 years) received less than 80% of the prescribed GH dose [18], and that adherence was positively correlated with height standard deviation score (SDS) (*P* < 0.001). Similarly, Desrosiers et al. reported that 9.5% (58/609) of patients on GH therapy missed more than half (>15) of their monthly injections [4]. Twelve-month height-velocity data in patients missing >15 injections/month (6.3 cm/year) was only 67% of the height velocity achieved by patients missing 11–15 doses/month (9.4 cm/year) (*P* ≤ 0.03). In the present study, reported overall compliance with treatment was good, with few injections missed. Care should be taken in interpreting these data, as not all patient diaries, which were used as the source of compliance data in the study, were returned for assessment.

The high patient acceptance of the device in this study, as well as ease of use, were aligned with a favorable safety profile and low reporting of technical complaints. Notably, patient satisfaction was particularly high among patients naïve to GH therapy. These patients also gave a positive assessment of the ease of injection and stated that they would like to continue use of the device in the future. Patient satisfaction with his/her GH device has been positively associated with adherence. In a study of 75 GH-deficient children, of a similar age (median (interquartile range) 12.3 (8.9  to  14.8) years) to those included in the present study, lack of choice of GH device was significantly (*P* < 0.05) associated with lower treatment concordance [17]. In an earlier study, concordance was reported to be unaffected by the choice of GH device [19]. In that study, following a change in clinic policy that allowed patients a free choice of GH device, there was no change in concordance with GH therapy. It should be noted, however, that concordance with GH therapy was already high at the clinic (87%). Interestingly, patients offered free choice of device cited better satisfaction with their treatment.

The current study was designed to evaluate patient's acceptance and satisfaction with NordiFlex and NordiFlex PenMate as well as to examine patient compliance with the device based on information recorded in patient diaries. No comparator devices were assessed directly; however, many patients had switched from other devices and therefore were able to indirectly compare their experiences with use of other pen devices. Analyses were made on a number of subgroups from within the patient population, for example, self-injectors versus those injected by parents/carers; treatment-naive versus nonnaive patients, to better understand the needs of different sectors of the patient population, and the benefits of the device and its accessory for these patients. 

In summary, in this study of patients injecting GH using NordiFlex PenMate, patients reported a good overall impression of the device, with patients and caregivers quickly learning to use the device, and the majority expressing a strong preference to continue using the device in the future. Physicians and nurses involved in training patients and caregivers in use of the device similarly considered the device to be easy to learn and to teach. Together, these findings support that NordiFlex PenMate is a well-accepted, preferred option for patients receiving daily subcutaneous injections of GH. 

The features of NordiFlex PenMate may simplify the injection process, help reduce pain perception, and, with this, facilitate patients' adherence to the regimen of daily injections, in turn improving their outcomes from GH therapy.

## Figures and Tables

**Table 1:** Patient demographic characteristics at baseline.

Characteristic	
Age (years), mean (SD)	11.27 ± 3.95
Sex (male/female), %	47/38 (55.4/44.6)
Height (cm), mean (SD)	139.5 (29.3)
Weight (kg), mean (SD)	36.3 (18.3)
Body mass index (kg/m^2^), mean (SD)	12.4 (4.9)
Diagnosis (*n*), (%)	
GHD	28 (32.9%)
Acquired GHD	9 (10.6%)
SGA	23 (27.0%)
Turner syndrome	11 (12.9%)
“Other”	14 (16.5%)
Previous GH therapy (*n*), (%)	
GH-naïve	19 (22.4%)
Switched from another GH device	66 (77.6%)

GH: growth hormone; GHD: growth hormone deficiency; SD: standard deviation; SGA: small for gestational age.

## References

[1] Farber RS, Kerrigan JR (2006). The multiple indications for growth hormone treatment of pediatric patients. *Pediatric Annals*.

[2] Drake WM, Howell SJ, Monson JP, Shalet SM (2001). Optimizing GH therapy in adults and children. *Endocrine Reviews*.

[3] Cutfield W, Lindberg A, Wikland KA, Chatelain P, Ranke MB, Wilton P (1999). Final height in idiopathic growth hormone deficiency: the KIGS experience. KIGS international board. *Acta Paediatrica, International Journal of Paediatrics, Supplement*.

[4] Desrosiers PM, O’Brien F, Blethen S (2005). Patient outcomes in the GHMonitor: the effect of delivery device on compliance and growth. *Pediatric Endocrinology Reviews*.

[5] Smith SL, Hindmarsh PC, Brook CGD (1993). Compliance with growth hormone treatment—are they getting it?. *Archives of Disease in Childhood*.

[6] Fidotti E (2001). A history of growth hormone injection devices. *Journal of Pediatric Endocrinology and Metabolism*.

[7] Ahmed SF, Smith WA, Blamires C (2008). Facilitating and understanding the family’s choice of injection device for growth hormone therapy by using conjoint analysis. *Archives of Disease in Childhood*.

[8] Dumas H, Panayiotopoulos P, Parker D, Pongpairochana V (2006). Understanding and meeting the needs of those using growth hormone injection devices. *BioMed Central Endocrine Disorders*.

[9] Gluckman PD, Cutfield WS (1991). Evaluation of a pen injector system for growth hormone treatment. *Archives of Disease in Childhood*.

[10] Müller J, Skakkebæk NE, Jacobsen BB (1999). Norditropin^®^ SimpleXx(TM): a liquid human growth hormone formulation, a pen system and an auto-insertion device. *Hormone Research*.

[11] Diglas J, Feinböck C, Winkler F (1999). Reduced pain perception with Pen Mate(TM), an automatic needle insertion device for use with an insulin pen. *Practical Diabetes International*.

[12] Oyarzabal M, Aliaga M, Chueca M, Echarte G, Ulied A (1998). Multicentre survey on compliance with growth hormone therapy: what can be improved?. *Acta Paediatrica*.

[13] Chan H (2001). Effects of injection duration on site-pain intensity and bruising associated with subcutaneous heparin. *Journal of Advanced Nursing*.

[14] Ida S, Yoshimura N, Nakacho M (2008). Evaluation of the use of PenNeedle® 32G Taper in children being treated with growth hormone. *Clinical Pediatric Endocrinology*.

[15] Stanhope R, Albanese A, Moyle L, Hamill G (1992). Optimum method for administration of biosynthetic human growth hormone: a randomised crossover trial of an auto injector and a pen injection system. *Archives of Disease in Childhood*.

[16] Main KM, Jørgensen JT, Hertel NT, Jensen S, Jakobsen L (1995). Automatic needle insertion diminishes pain during growth hormone injection. *Acta Paediatrica*.

[17] Kapoor RR, Burke SA, Sparrow SE (2008). Monitoring of concordance in growth hormone therapy. *Archives of Disease in Childhood*.

[18] Hunter I, de Vries C, MacDonald TMA, Greene S (2000). Human growth hormone therapy: poor adherence equals poor growth. *Archives of Disease in Childhood*.

[19] Wickramasuriya BPN, Casey A, Akhtar S (2006). Factors determining patient choice of device for GH therapy. *Hormone Research*.

